# Anti-EGFR anchored paclitaxel loaded PLGA nanoparticles for the treatment of triple negative breast cancer. *In-vitro* and *in-vivo* anticancer activities

**DOI:** 10.1371/journal.pone.0206109

**Published:** 2018-11-08

**Authors:** Vijayan Venugopal, Shalini Krishnan, Vasanth Raj Palanimuthu, Subin Sankarankutty, Jayaraja Kumar Kalaimani, Sundram Karupiah, Ng Siew Kit, Tang Thean Hock

**Affiliations:** 1 Department of Pharmaceutical Technology, Faculty of Pharmacy, Asian Institute of Medical Science and Technology (AIMST) University, Kedah, Malaysia; 2 School of Pharmacy, Medical Biology Centre, Queen's University Belfast, Northern Ireland, UK; 3 China Medical University—Queen's University Belfast joint college (CQC), Shenyang, China; 4 Department of Pharmaceutical Chemistry, Faculty of Pharmacy, Asian Institute of Medical Science and Technology (AIMST) University, Kedah, Malaysia; 5 Advanced Medical & Dental Institute, Universiti Sains Malaysia, Penang, Malaysia; Brandeis University, UNITED STATES

## Abstract

The aim of the present study is to analyze the viability of anti-EGFR anchored immunonanoparticle (INP) bearing Paclitaxel (PTX) to specifically bind the EGFR protein on the TNBC cells. The NP was prepared by nanoprecipitation and characterized the particle size, charge, entrapment of drug and release of it. The anti-EGFR anchored and the integrity was confirmed by SDS-PAGE. Cytotoxicity and NPs cellular uptake was analyzed with MDA-MB-468 type cancer cells and the EGFR expression was confirmed by PCR, qualitatively and quantitatively. The *in-vivo* antitumor activity of INP was determined by using athymic mice model and targeting efficiency was measured by calculating the PTX accumulation in the tumor plasma. The prepared INP with the size of 336.3 nm and the charge of -3.48 mV showed sustained drug release upto 48 h. The INP showed significant reduction of cancer cell viability of 10.6% for 48 h with 93 fold higher PTX accumulation in the tumor plasma compared with NPs. Based on these reports, we recommend that anti-EGFR anchored PTX loaded NP may have the ability to target the TNBC cells and improve the therapeutic action and subsidize the side effects of PTX for the treatment of TNBC.

## 1. Introduction

Triple negative breast cancer (TNBC) is an aggressive type of heterogeneous phenotype based diseases, which are specified by absences of receptors expressions such as Progesterone Receptor (PR), Estrogen Receptor (ER), and Human Epidermal Growth Factor Receptor 2 (HER2) [1]. Currently, chemotherapy is the only option to treat TNBC. However, it is ineffective, due to the lack of receptor expression for chemotherapy. TNBC can be very aggressive, more difficult to cure and more likely to spread and recur.

Paclitaxel (PTX) is the best microtubule stabilized drug which was approved by the USFDA for the treatment of various cancers like ovarian, breast, lung, Kaposi's sarcoma and also cervical, prostate, head and neck cancer. PTX chemotherapy-based regimens have been routinely used in the Neoadjuvant setting for TNBC [2]. PTX shows significant effects on mitosis that are efficient to kill the cancer cells on interphase of the cell cycle. PTX concentration (50–1000 fold) varies depends on site and cancer type, treatment schedule and accumulation index. Therefore the concentration of PTX is almost certainly higher in the tumor than in the plasma. However, it can cause serious adverse effects like nephrotoxicity and neurotoxicity and other problems with chemotherapy associated with drug resistance. In order to reduce the side effects of drugs, it is imperative that the dose and dosage form(s) are carefully designed.

The breast cancer stage, as well as the tumor grade, will influence the cancer prognosis [3]. Immuno-histochemical analysis evidence that TNBC is analogous of high expression of the proliferation bio-markers such as Ki-67, cyclin E, mutated p53, Epidermal Growth Factor Receptor (EGFR), P-cadherin, vimentin, and mutated BRCA1, CK5/6, c-KIT in all the populations [4–6]. However, there is evidence that Basal-Like Breast Cancer (BLBC) and TNBC might be two different biological entities [7]. Studies have been delineating that, 80% of the tumor that expresses EGFR basal marker in a western population [8–11]. EGFR is a member of ErbB family and the protein comprises of following domains such as extracellular binding ligand, transmembrane and cytoplasmic tyrosine kinase. Frequently EGFR gene is mutated and overexpressed in head, lung, neck, colon, pancreatic, brain and breast cancers especially in TNBC by stimulating the tumor progression. Consequently, EGFR is a fascinating drug target to inhibit EGFR expression by tyrosine kinase inhibitors (TKIs) and mAbs. The anticancer activity of PTX in prototype of TNBC, we hypothesize that PTX-entrapped NPs anchored with anti-EGFR to have therapeutic action in TNBC.

## 2. Materials and methods

### 2.1. Materials

PLGA-PEG polymer was purchased from Advanced Polymeric Materials lnc, Canada. Paclitaxel (PTX) was purchased from Chemtron Biotechnology Sdn Bhd, Malaysia Anti-EGFR was purchased from Thermo Scientific, USA. Bovine Serum Albumin (BSA), Fetal bovine serum (FBS), Ponceau red stain, TRIZOL were procured from Sigma Aldrich, USA. MDA-MB- 468 Cell lines and Leibovitz's L-15 medium (Catalog Number: C0006-01, Part No: C0006003) were source from AddexBio, USA and supplied by BioREV Sdn Bhd, Malaysia. Superscript 1V RT kit and Fluorescein isothiocyanate (FITC) were obtain from Thermo Fisher Scientific corporation (USA), Ripa Lysis Buffer and PCR mix were purchased from Merck Millipore, USA and all other reagents & solvents used were analytical grade.

### 2.2. Methods

#### 2.2.1. Preparation of PTX loaded nanoparticle (NPs)

NP was formulated by nanoprecipitation method [12]. PTX and PLGA-PEG polymer were dissolved in 5 ml of Dichloro Methane (DCM). The homogeneous mixture was added dropwise to double the volume of distilled water under ultra-probe sonicator (QSonica, USA) at 60 kHz frequency for 2 minutes. The NPs were precipitated in the non-solvent system (water). The resulting precipitated suspension was stirred uncovered for 8 h at room temperature. The formed NPs were washed by centrifugation at 1000 gm for 30 minutes. The prepared NP was suspended in water and centrifuged to detach the unentrapped PTX. Finally, the NP was freeze-dried at -20˚C to obtain dry powder form of the NPs.

#### 2.2.2. Preparation of anti-EGFR protein anchored nanoparticle (INP)

The anti-EGFR protein was anchored on the surface of the NP by a cross-linking method using the cross-linking agent m-maleimidobenzoyl-N-hydroxysuccinimide ester (MBS) (28). Initially, anti-EGFR protein was thiolated by treated with 100 μl anti-EGFR protein solution (1mg/ ml of phosphate buffer saline pH of 8.0) incubated with MBS solution (5.7mg in 5.0 ml phosphate buffer, pH 8.0), for 3 h at 20° C under constant magnetic stirring. Then, the NP surface was activated with an MBS. Briefly, 5 mg of NPs were dissolved in 2 ml PBS (pH 8.0) and 1ml of MBS (3.14 mg per ml of DMSO) magnetic stirred for 1 hour at 20° C. To anchor the anti-EGFR protein on the surface of activated PLGA-PEG NPs, both the activated solution was mixed together by magnetic stirring for 3 h at 10° C to produced INPs.

#### 2.2.3. Physiochemical characterization of the PTX-NPs

The nanoparticle size, Polydispersity index (PDI) and zeta potential were analyzed by using Zetasizer 300 HS type equipment (Malvern Instruments, UK). The required quantity of the sample was dispersed in distilled water and the size of NPs was measured at 25˚C. Based on the intensity of light scattered from the NPs, the diameter of the particle was calculated. All the parameters were measured in triplicate. Entrapment efficiency of NPs was analyzed to determine the entrapment efficiency. About 2 mg of prepared NPs were solubilized in 5 ml of DCM. The suspension was centrifuged at 10000 rpm for 15 minutes. The drug concentration was analyzed by RP-HPLC (Shimadzu, Japan) at 227 nm. The morphology of the NPs was analyzed by Transmission Electron Microscope (TEM). A pinch of NPs sample was spread on a metal stub. The meal stub was coated with gold by Hitachi 1010 ion sputter and the morphology was observed under Hitachi 3000 N TEM (JSM 5610 LV SEM, JEOL, Japan) chamber. The NPs image was snapped at 20 kV with a chamber pressure of 0.6 mm Hg. All the data in the study was expressed as Mean ± SD of triplicate readings using XLSTAT 2015.1.

#### 2.2.4. *In-vitro* release study

The *in-vitro* release study of NP was carried out by employing modified Franz diffusion apparatus at 37± 2°C. About 20 mg drug equivalent NPs were suspended in the donor compartment containing pH 7.4 phosphate buffer. The drug release was quantified by periodical sampling (5ml) from the receptor medium and the fresh phosphate buffer saline pH 7.4 was replaced. The samples were filtered through the membrane filter (0.22μ) and the quantity of drug released was computed by an RP-HPLC method at 227 nm. Based on the *in-vitro* release data, the values were subjected to zero order, first order, Higuchi, Peppas, and Hixson release kinetics formula to determine the mechanism of drug release from the NPs.

#### 2.2.5. SDS-PAGE gel electrophoresis analysis

The structural integrity of anti-EGFR protein after being anchored onto the NP surface was analyzed by SDS-PAGE gel electrophoresis and comparison with control (pure anti-EGFR protein). The gel electrophoresis was conducted under conditions of constant current supply at 125 V in a Tris/glycine/SDS buffer.

#### 2.2.6. Cytotoxicity studies

The viability of cancer cells was calculated by MTT method using MDA-MB-468 TNBC cell line. The cells were cultivated using Leibovitz's L-15 Medium supplemented with 10% FBS (heat inactivated), 1% antibiotic and antimycotic solution. The cells were kept at 37^o^ C, in a humidified 5% CO_2_ incubator. About 2 X 10^5^ cells were cultured in the T25 flask and the modified protocol from Denizot and Lang 1986 was followed for the cell viability experiment by MTT assay [13]. Briefly, 2 x 10^4^ cells were transferred in 96 well plates and the cells were incubated for attachment at proper conditions for 24 h. To determine the IC_50_ value of PTX, the cells were incubated with different concentrations (0.02, 0.2, 2, 20 μg/ml) of PTX. After 12 h of incubation time, the medium was replaced with 10μl of MTT reagent (2mg/ml) in PBS for 4 h at 37° C to produce formazan. The pink color formazan produced by viable cells were dissolved in 50 μl isopropanol. The plates were shaken for 20 minutes and the absorbance was measured at 640 nm using microplate reader (Elisa reader, BioTek XL-800) and percentage cell viability was calculated. Based on this result, the IC_50_ value of PTX was calculated by using Microsoft Excel 2010. An equivalent quantity of IC_50_ concentration of PTX-NP, INP, and plain NP was subjected to the similar procedure of MTT assay was followed for 24 h and 48 h incubation and the results were compared with blank and control.

#### 2.2.7. Nanoparticles cell uptake

To study the NP cellular uptake, The MDA-MB-468 cells were cultured in 6-well plate at the count of 2 ×10^5^ cells/well and the cells were incubated with PTX-NP and INP were tacked fluoro isothiocyanate (FITC) and stored for 24 h. Later, the cells were washed four times with phosphate buffer solution to remove the NPs which could not enter the cells. Finally, the cells were stabilized with a drop of ethanol and incubated for 20 min. The cells were observed under fluorescence microscopy to detect the uptake of NPs.

#### 2.2.8. DAPI staining

Apoptosis of MDA-MD-468 cell line was morphologically identified by 4,6-diamidino-2-phenylindole (DAPI) staining. The MDA-MD-468 cells were seeded in 6-well plates containing a coverslip with 1 x 10^5^ cells per well and cultured at 37°C for 24 h. Subsequently, the cells were treated with PTX-NP and INP for 48 h. The cells were fixed with 4% paraformaldehyde in PBS at room temperature for 15 min and then, stained with 0.2 μg/ml DAPI in PBS at dark room for 20 min and washed the cells twice with PBS. The mounted the coverslips and observed under the fluorescent microscope with a 340/380nm excitation filter.

#### 2.2.9. RNA extraction and reverse transcriptase PCR

MDA-MB-468 breast cancer cells were cultured in 6-well plates and exposed to PTX-NP and INP for 24 h. Total RNA was extracted from the cultured cells using a Trizol reagent (Invitrogen, Carlsbad, CA) according to the manufacturer’s instructions. First-strand cDNA was synthesized using the SuperScript IV RT Kit as per the manufacturer’s instructions (Thermo Fisher Scientific). Two microliters of template cDNA were added to the final volume of 20 μl of reaction mixture. Reverse transcriptase PCR cycle parameters included 1 min at 95 °C followed by denaturation at 94°C for 30 s, annealing at 58 °C for 60 s and extension at 35 cycles of 72 °C for 60 s, and final elongation at 72 °C for 10 minutes. The sequences of the specific sets of primer for native EGFR, NP and INP treated cells expressed EGFR were analysed in this study. The cDNA samples were subsequently amplified and quantified using the Gel Doc XR^+^ imaging system with EFGR primers (5′- TGCCCATGAGAAATTTACAGG -3′and 5′- ATGTTGCTGAGAAAGTCACTGC -3′). The PCR product of EGFR protein cDNA was run by agarose gel electrophoresis and the band was imaged by using Bio-Rad Gel Doc XRSystem.

#### 2.2.10. *In-vivo* evaluation studies

Twenty athymic female mice (6 weeks matured) were purchased from USM laboratories, Penang, Malaysia and caged under laminar flow controlled pathogen-free air at on/off dark and light cycles, and maintained with food and water ad libitum. About 0.1 ml sample containing 2×10^5^ MDA-MB-468 cells were injected into the right flank of mice. Tumor volume was measured once every week using an electronic caliper until the volume reached 120mm^3^ (7 days post injection). The tumor volume was estimated using the formula of an ellipsoid (length × width^2^ × 0.5236). The study was approved by the Human and Animal Ethics Committee (AUHACE/FOP/2015/11) of AIMST University and the study was conducted according to Animal Research Review Panel guidelines.

The animals were divided into four groups each contains 5 mice. Each group was treated by the following injections. Group 1: PTX intravenous injection (2 mg/kg); Group 2: PLGA-PEG polymer solution (control); Group 3: PTX-NP (dose equivalent to 2 mg/kg) and Group 4: INP (dose equivalent to 2 mg/kg). Every 24 h, the tumor volume was measured for anti-tumor activity. At the end of experiment, the mice were sacrificed with isofluorane anesthetic. The tumor were collected and preserved at -70° C, later tumor samples were homogenized by Ultra-probe sonicator (Qsonica, USA) with ethyl acetate as extracting solvent. The precipitated PTX was calculated by RP-HPLC method.

#### 2.2.11. RP-HPLC analysis of plasma PTX concentration

The tumor PTX concentration was analysed by using RP-HPLC method. 200 μl of whole blood was collected periodically at 1, 2, 4, 8, 12 & 24 h in the heparin coated tubes. The blood samples were centrifuged at 4° C for 15 minutes at 8000 rpm, and the separated plasma was stored at -80° C for further analysis. PTX was extracted by using liquid-liquid extraction technique using solvent system (tetra butyl methyl ether: diethyl ether in the ratio of 50:50, v/v). Plasma samples (0.1 mL) were spiked with 1 mL of extracting solvents by high speed vortex mixer for 2 minutes, later centrifuged at 4°C for 15 minutes at 5000 rpm to isolate the layers (aqueous and organic layers). The organic layer was evaporated under nitrogen condition to get residual PTX. The residue PTX was dissolved in 200 ml of HPLC mobile phase containing methanol: water: TFA in the ratio of 85: 15: 0.1, v/v/ and sonicated for 20 seconds. The 20 μL of reconstituted solution of PTX was injected into the HPLC column in the flow rate of 1.0 mL / minute and PTX was analysed at the wavelength of 225 nm [14].

#### 2.2.12. Bio-distribution and targeting efficiency

Bio-distribution and targeting efficiency of INP were evaluated by *in-vivo* animal fluorescence imager and the image was compared with PTX-NP and PTX groups. FITC loaded INP and PTX-NP were injected into anesthetized tumor-induced mice. After 24 hours of administration, the mice were subjected to *in-vivo* fluorescence imaging system (Kodak *In-vivo* FX Imaging Station, USA). The images were snapped at one-second exposure and fluorescence intensity were determined.

### 2.3. Statistical analysis

Analysis of variance (ANOVA) and linear regression analysis was performed on MTT assay by two tailed student t-test. The *in-vivo* anti-tumour activity was analysis by two-way ANOVA method. Means (n = 3) were compared Bonferroni post-tests using Graph Pad Prism software version 7.0. A value of p<0.05 consider to be significant.

## 3. Results

### 3.1. Preparation and characterization of NPs

**Fig 1:**
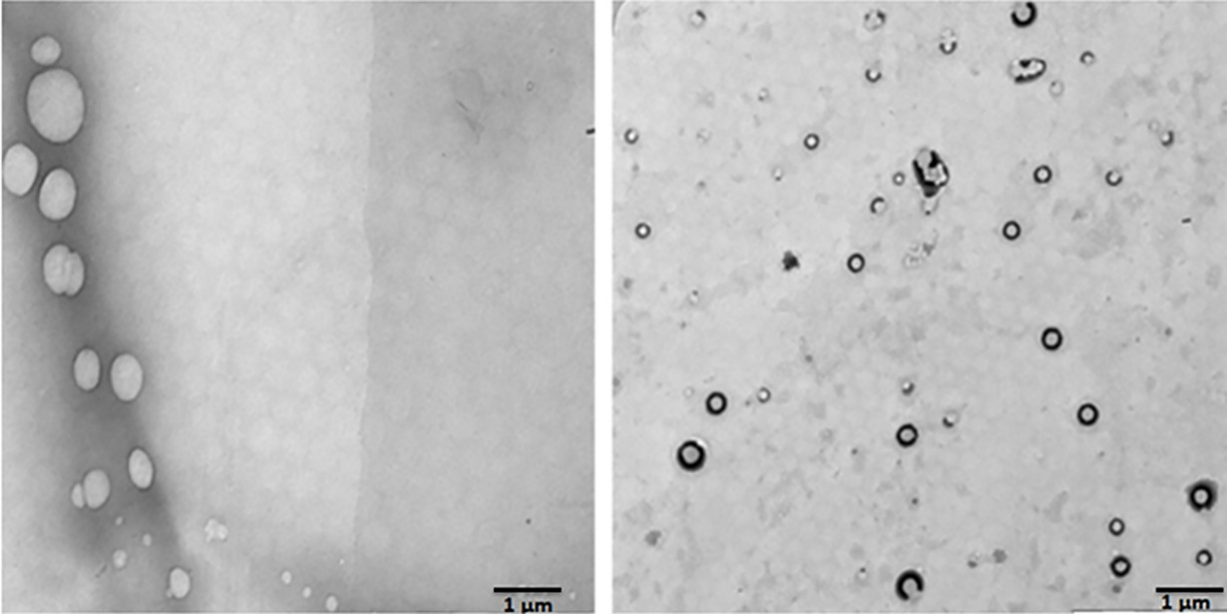
Transmission electron microscopic (TEM) images of nanoparticle. (Left side) NP, Right side is a TEM image of INP. An inserted picture showed the appearance of smooth surfaces with spherical shape nanoparticle.

**Fig 2:**
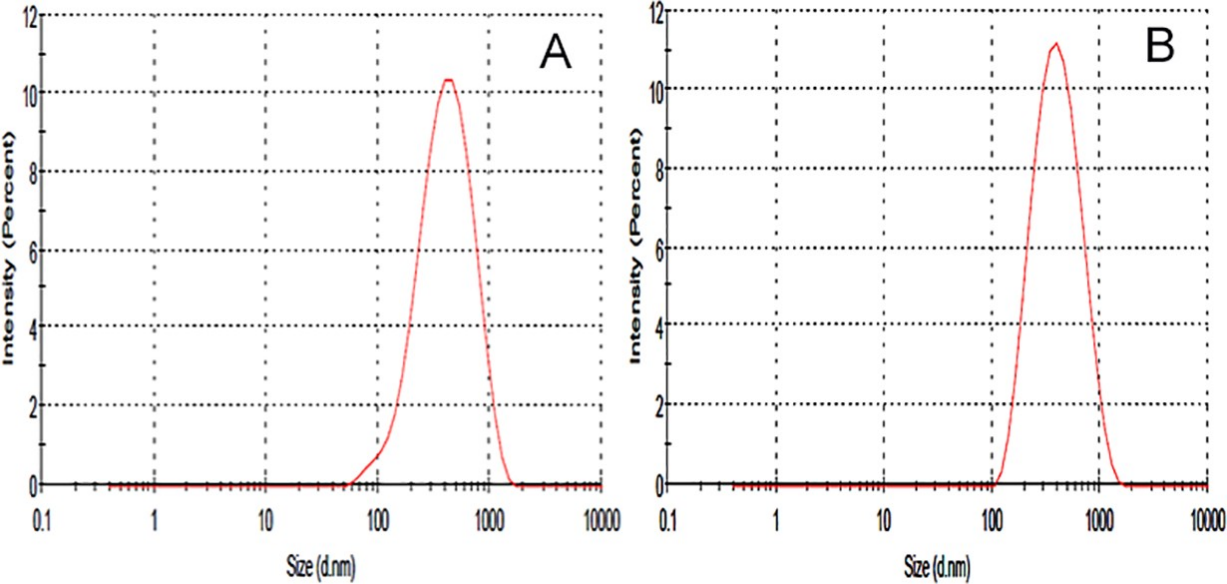
Particle size graph of NP (A), INP (B).

**Fig 3:**
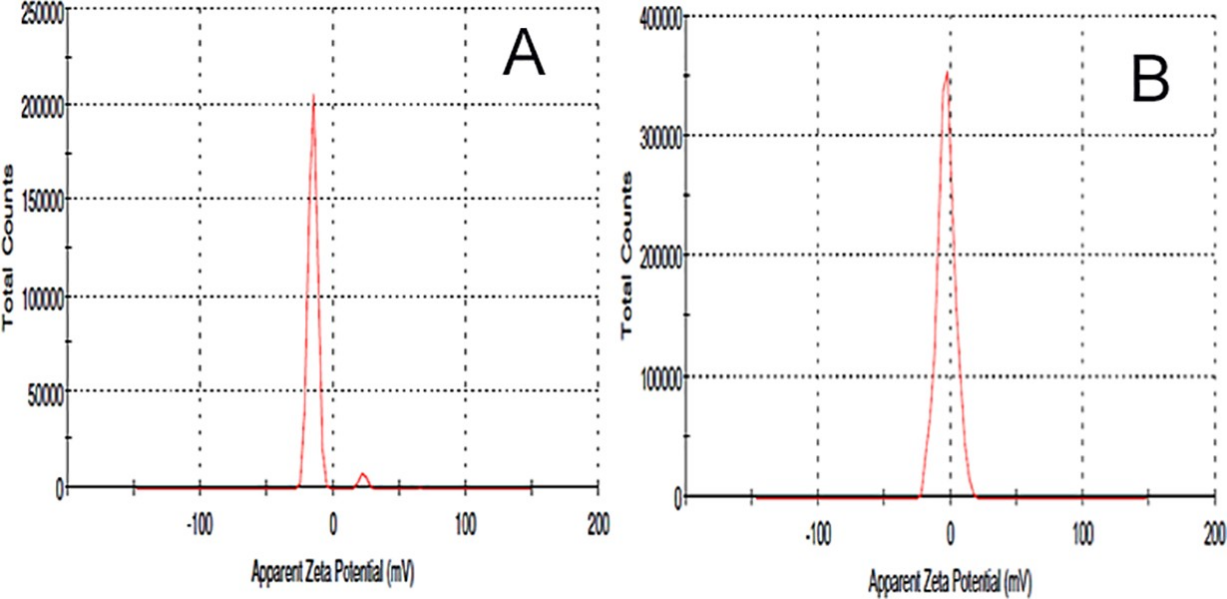
Zeta potential of NP (A), INP (B).

The NPs were formulated by nanoprecipitation method by utilizing the ouzo effect. This effect helps the organic solvents partitioned in an aqueous solvent as a nanosized particle. The NP shape and surface were analyzed by TEM imaging studies (Fig 1). The NP shows smooth and spherical shape, whereas, INP shows smooth and spherical shape with a thick surface due to the coating of anti-EGFR. Particle size, PDI and zeta potential of the samples were investigated using Zetasizer. NP size is the key factor for the drug diffusion and permeation for cell-membrane to biological systems. The NP and INP particle size were 317.5±1.4 nm 335.3±2.2 nm respectively (Fig 2). The NP size of all the formulations shows nanometer range with mono-dispersion with the polydispersity index (PDI) of 0.3 and 0.26 for the NP and INP respectively. The zeta potential of the NP is an important factor for drug permeation and stability (particle aggregation). The zeta potential expresses the particle-particle aggregation and repulsion action. The zeta potential of NP and INP were -12.7±0.22 mV and -3.48±1.2 mV respectively (Fig 3), which indicates that the formulations were more stable with less aggregation. The negative charges of NPs were exhibited, due to the presences of the carboxylic group on the NPs surface [15]. The entrapment efficiency of NP was analyzed by ultracentrifugation technique and the PTX concentration was measured by HPLC method. The EE of NP and INP were 85.42 ± 2.6% and 85.58 ± 1.2% respectively.

**Fig 4:**
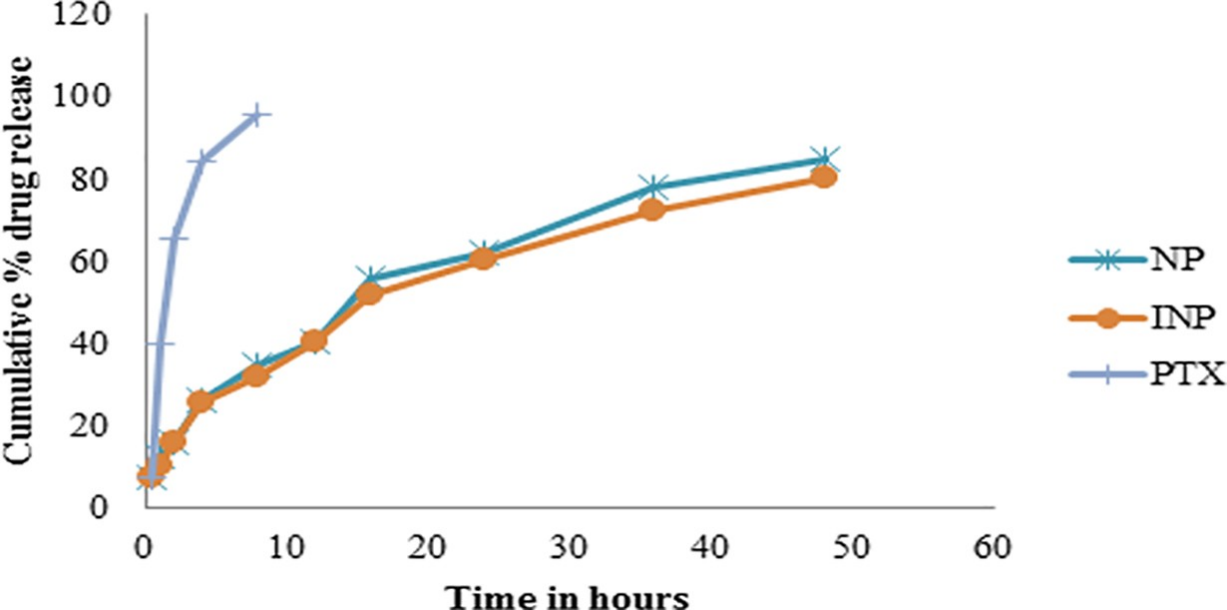
*In-vitro* drug release studies of pure PTX, NP and INP.

The NP and INP showed the drug release upto 48 h in the range of 7.62 ± 0.8% to 85.14 ± 1.2% and 7.4 ± 0.2% to 80.24 ± 3.2% respectively (Fig 4). Based on the drug’s release character, the kinetics of drug releases was calculated. Both the nanoformulation followed the zero order with Higuchi type of drug releases. Initially, the formulations showed burst drug release in the range of 7%. From this, it was proved that the low drug releases was due to the formation of more polymer layer around the drug and the drug molecules were cross-links between the lacto-glycolic acid linkages and acquired the sustained release of drugs for an extended period of time [16]. At the end of the 48 h, a narrow percentage of the drug was released from NP and INP 85.14 ± 1.2%, 80.24 ± 3.2% respectively. The kinetics of drug release of NP and INP formulations followed zero order of R2 = 0.9325 and 0.9453 and Higuchi of R^2^ = 0.9275 and 0.9131 respectively. The kinetics model expressed that both the formulations significantly followed the sustained diffusional controlled release of drug from the matrix of nanoparticle.

**Fig 5:**
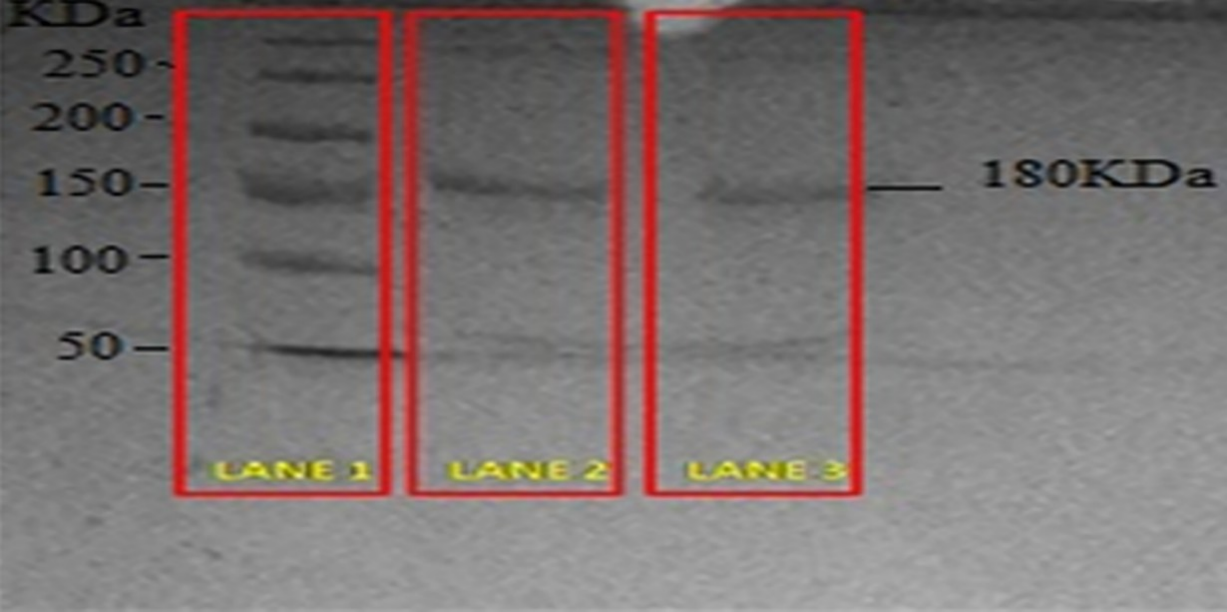
SDS-PAGE gel electrophoresis image anti-EGFR. Lane 1: ladder, Lane 2: Pure anti-EGFR, Lane 3: Immunonanoparticle.

NP anchored anti-EGFR structural integrity was analyzed by using SDS-PAGE gel electrophoresis. The pure anti-EGFR protein band and INP anchored anti-EGFR band were similar. The comparison of pure anti-EGFR remains the same after being interlocked on the NP surface (Fig 5).

### 3.2. *In-Vitro* anti-cancer studies

**Fig 6:**
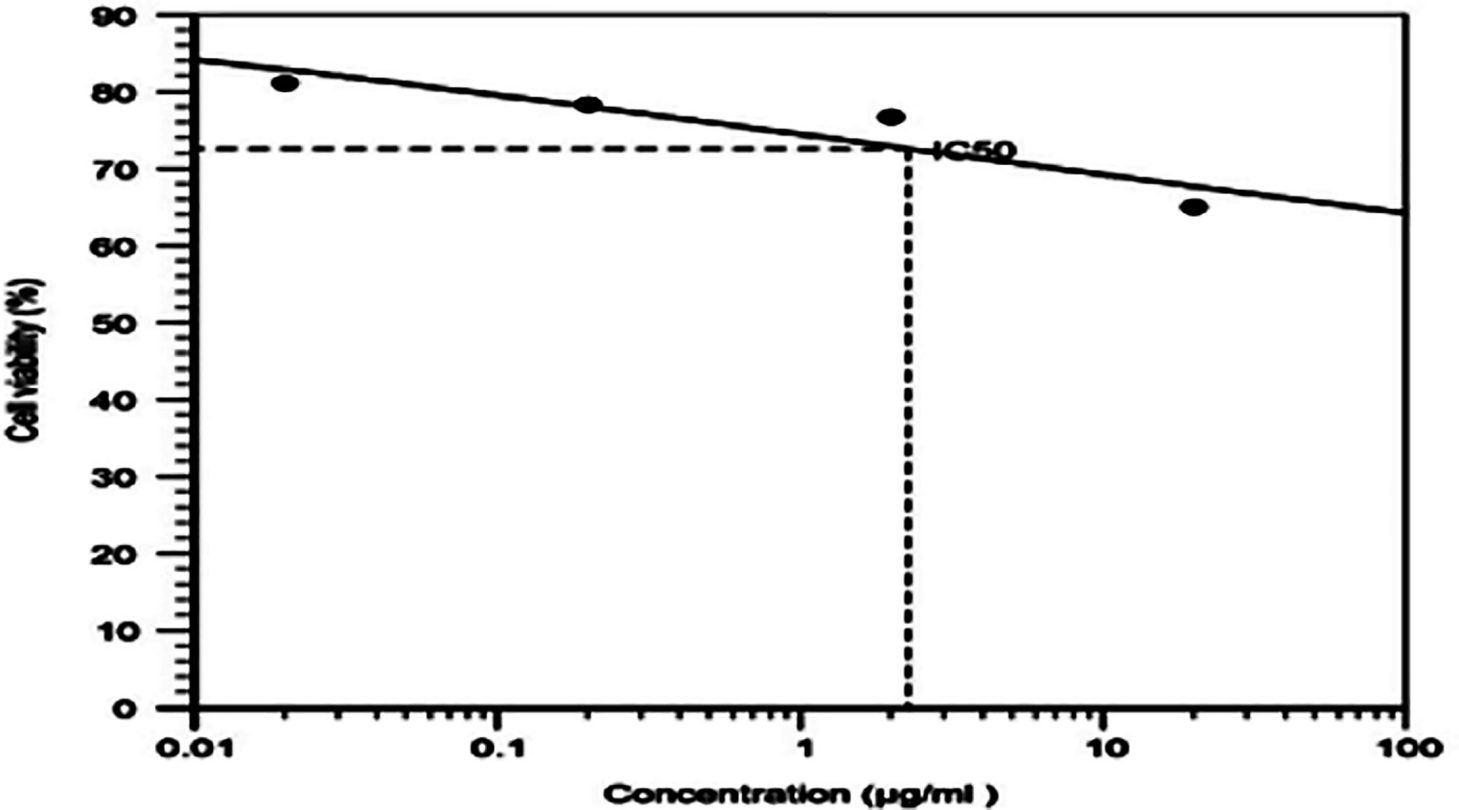
Cell viability studies of different concentration of PTX incubated in MDA-MB-468 breast cancer cell line. “The cancer cells were incubated with 0.02, 0.2, 2, 20 μg/ml concentrations of PTX. The cell viability was studied by MTT assay and values were compared with control (without treatment). The values were obtained by mean ± standard deviation (n = 3) independent experiments in the triplicate manner.

The cytotoxicity of PTX, NP and INP were characterized by MTT assay method using MDA-MB-468 breast cancer cells. Initially, the IC_50_ value of PTX was determined by the various concentrations of PTX and it was found to be 2.27 ± 0.2μg/ml (Fig 6).

**Fig 7:**
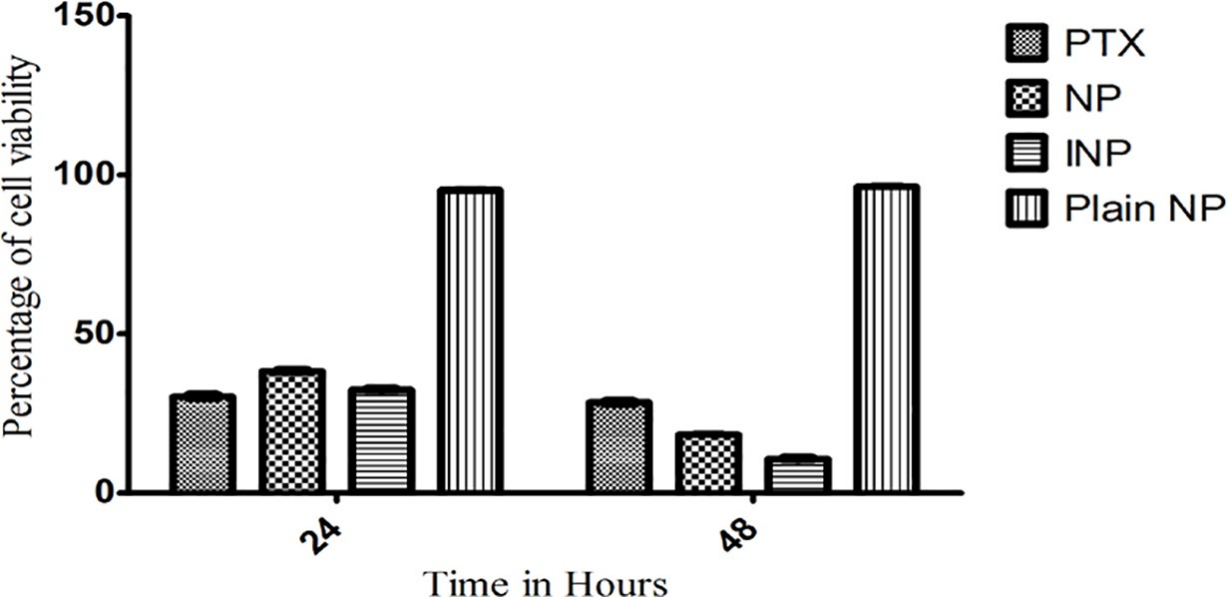
Cell viability studies of NP and INP, PTX and Plain NP, incubated with MDA-MB-468 breast cancer cells for 24 and 48 hours. “The cell viability was studied by MTT assay using 96 well plates and values were compared with blank nanoparticles and control (PTX solution Analysis of variance (ANOVA) and linear regression analysis was performed on the tumor volume. Means (n = 3) values were compared with two way ANOVA test. Means (n = 3) values were compared with two tailed student t test. NPs and INP showed statistical differences of ***P<0.001 as considered more significant than PTX”.

Furthermore, PTX, NP and INP, as well as plain NPs were incubated with the TNBC cells for 24 and 48 h. The plain NP showed cell viability in the range of 95.23 ± 2.4 and 96.2 ±1.8% in 24 and 48 h respectively (Fig 7). The results showed that PLGA and copolymer exhibit cytotoxicity on cancer cells, it could be equal to the 0.002 μg/ml concentration of PTX. NP showed the significant cytotoxic activity of 32.8 ±1.8% and 18.2±1.6% at 24 h and 48 h respectively (Fig 7) and the INP showed significant cytotoxicity. i.e, remarkable reduction of cell viability 32.4 ± 2.2% and 10.6 ± 3.4% (2 μg/ml of PTX) at 24 and 48 h respectively.

**Fig 8:**
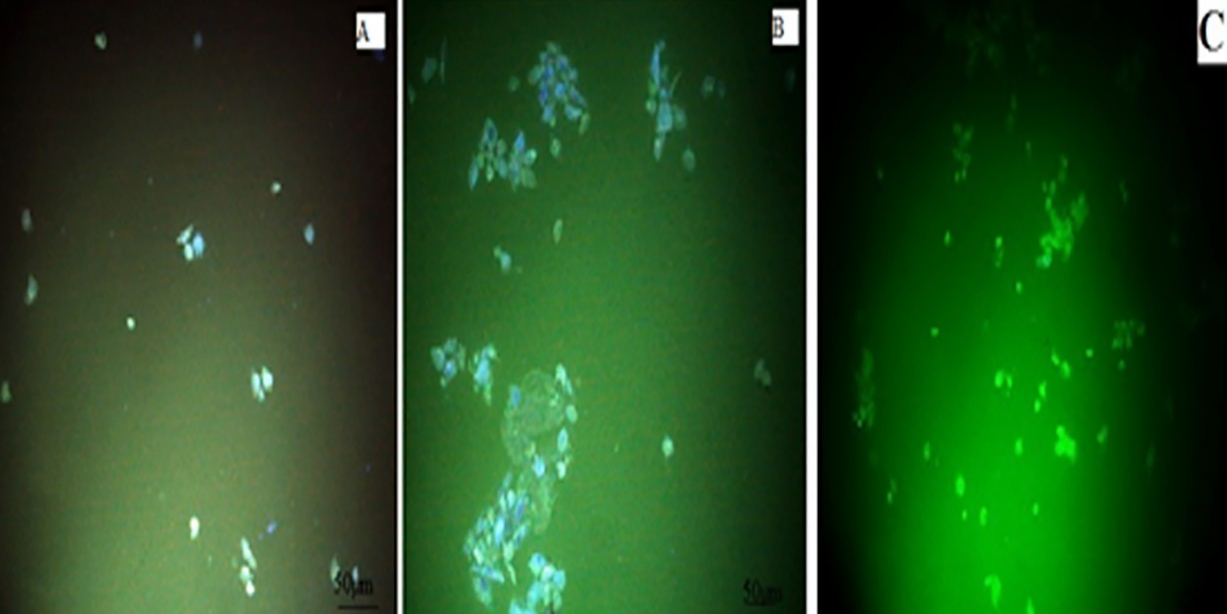
Fluorescence’s microscopic images of A) NP, B) INP, C) Untreated MDA-MB-468 cells. Both the formulations showed significant uptake of nanoparticle into the cells (green color fluorescence showed inside of the cells, which indicated that nanoparticles were penetrated into the cells).

The NPs cellular uptake was internalized in cancer cells, the cellular uptake of NPs and INP were anchored FITC and incubated with MDA-MB-468 cells for 24 hours. The fluorescence images of NP and INP were compared with untreated cells. The images show FITC tacked NPs (Green) bound in the cellular constituents of TNBC cells. The pictures (Fig 8) clearly show that NPs were penetrated into the cell membrane and showed significant cellular uptake by the fluorescence emission from the cells.

**Fig 9:**
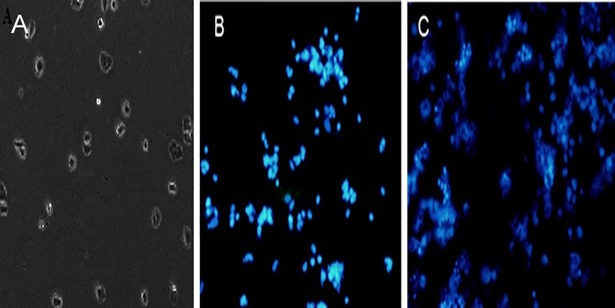
DAPI staining of MDA-MB-468 cells after incubation for 48 h of (A) Untreated cells (Control), (B) PTX-NP (C) INP.

DAPI is the most popular nuclear counterstain. The nuclear fragmentation which induced by the nanoparticles was observed by DAPI staining under a fluorescence microscope. The untreated MDA-MD-468 cells showed homogenous nuclei (smooth nuclear) with no evidence of segmentation and fragmentation. Whereas, cells treated with PTX-NP and INP were showed significant nuclear staining (Fig 9).

**Fig 10:**
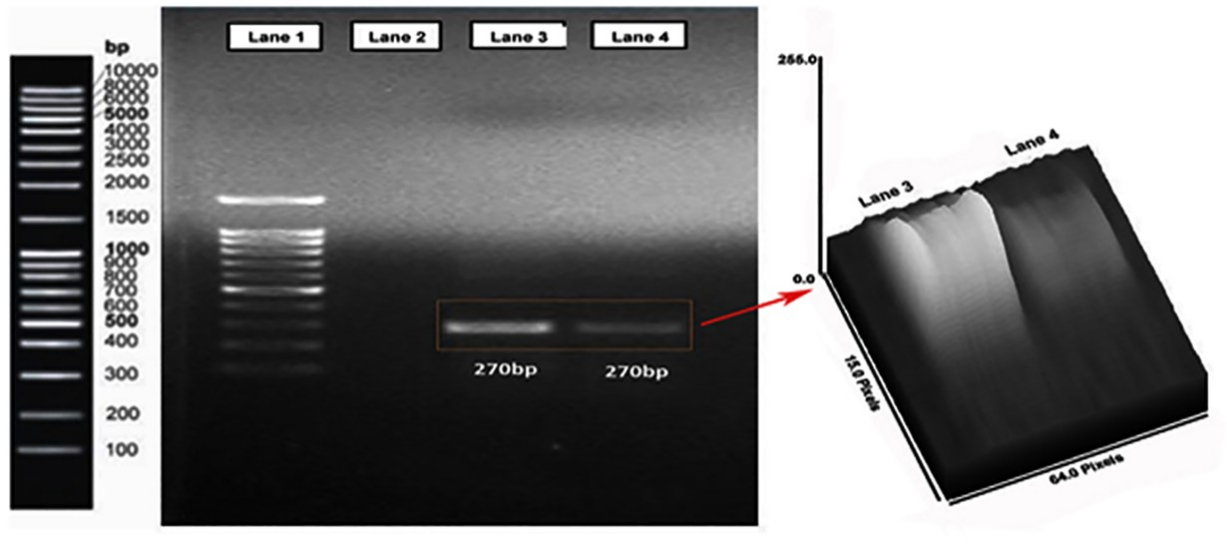
Agarose gel electrophoresis analyzed an image of the amplified EGFR gene expression cDNA. Lane 1 gene marker ladder, Lane 2 is control (Water), Lane 3 showed the band for PTX loaded nanoparticle treated TNBC cells. Lane 4 showed immunonanoparticle (INP) treated TNBC cells. It’s proven that the intensity of EGFR expression was declined with INP than NPs.

The EGFR gene expression could be estimated quantitatively and qualitatively by PCR amplified EGFR followed by agarose gel electrophoresis. The MDA-MB-468 cells were incubated with NP and INP and it was subjected to agarose gel electrophoresis and band has an amplicon size of 270 bp, it confirmed the EGFR gene expression (Fig 10).

### 3.3. *In-vivo* anti-tumor activity

**Fig 11:**
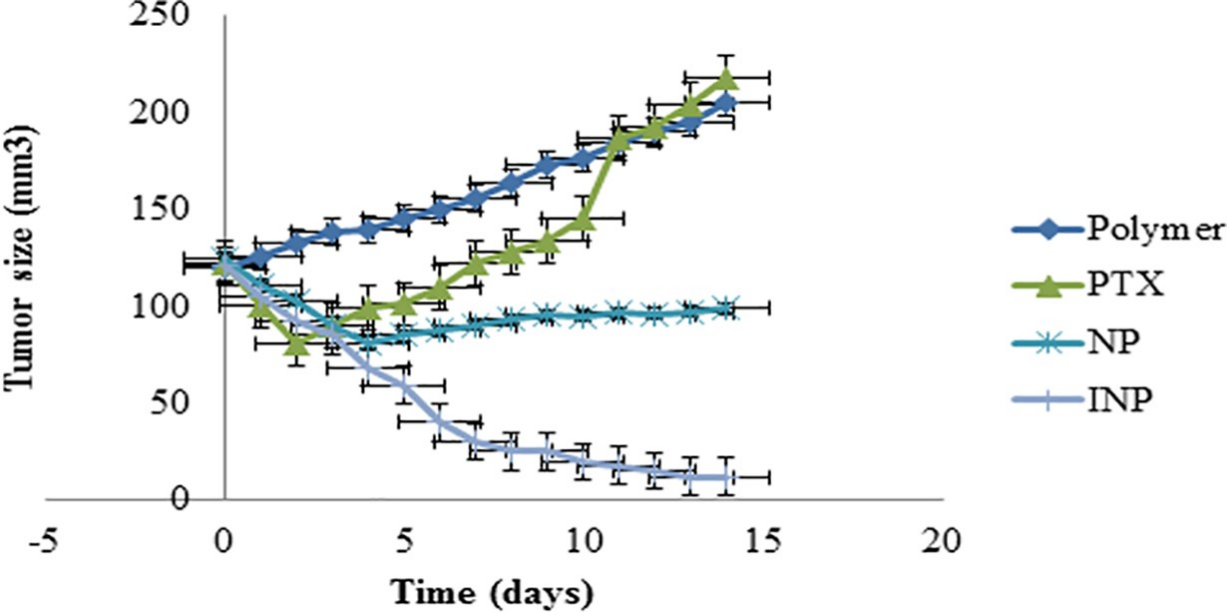
*In-vivo* tumor inhibition study in mice, tumor was induced by injecting MDA-MB-468 cells. The antitumor activity of NP and INP were significantly inhibited compared with PTX (control), polymer only. The highest tumor inhibition was observed in mice treated with INP*** than NP***. Analysis of variance (ANOVA) and linear regression analysis was performed on the tumor volume. Means (n = 3) values were compared with two tailed student t test. NPs and INP showed statistical differences of ***P<0.001 as considered more significant than PTX”.

The *in-vivo* antitumor activity of INP and NP were investigated in athymic female mice. The tumor was induced by injecting MBA-MB-468 cells, and tumor volume reached to 120 mm^3^, after treatment of formulations, tumor volume was measured until 14 days. Throughout the experiment, animal body weight and death were monitored and found that no changes in body weight. The mice treated with PTX equivalent of 2mg / kg of INP showed significant tumor reduction than NP. The (Fig 11) showed that INP having the highest degree of tumor reduction than other formulations and the statistical analysis evidenced that difference was p<0.001 than NP and PTX groups for all the 14 days. These data argue that anchoring of anti-EGFR targeted nanoparticles enhance the antitumor action by specifically binding with the EGFR protein; expressed by TNBC, result in significant shrinkage of tumor over the time. Taken together that, the results showed that INP may be successful way to target and treat TNBC without doubts.

### 3.4. PTX distribution on tumor treated animal plasma

**Fig 12:**
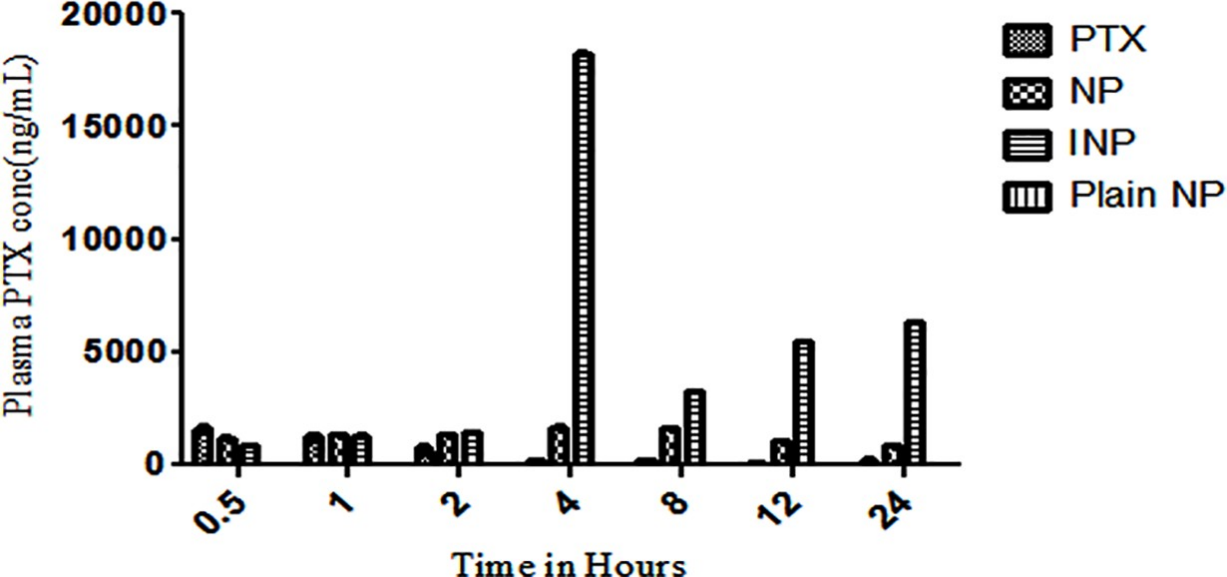
Analysis of Plasma PTX concentration in mice tumors. PTX plasma concentration of athymic mice treated with plain NP, PTX, and PTX loaded NP and anti-EGFR targeted PTX NP (INP) determined by RP-HPLC method. Samples were collected at different points up to 24 h and extracted the PTX. N = 3 per treatment mice. Data were shown ± SD, NPs and INP were showed ***p < 0.001, consider being significant compared with PTX.

**Fig 13:**
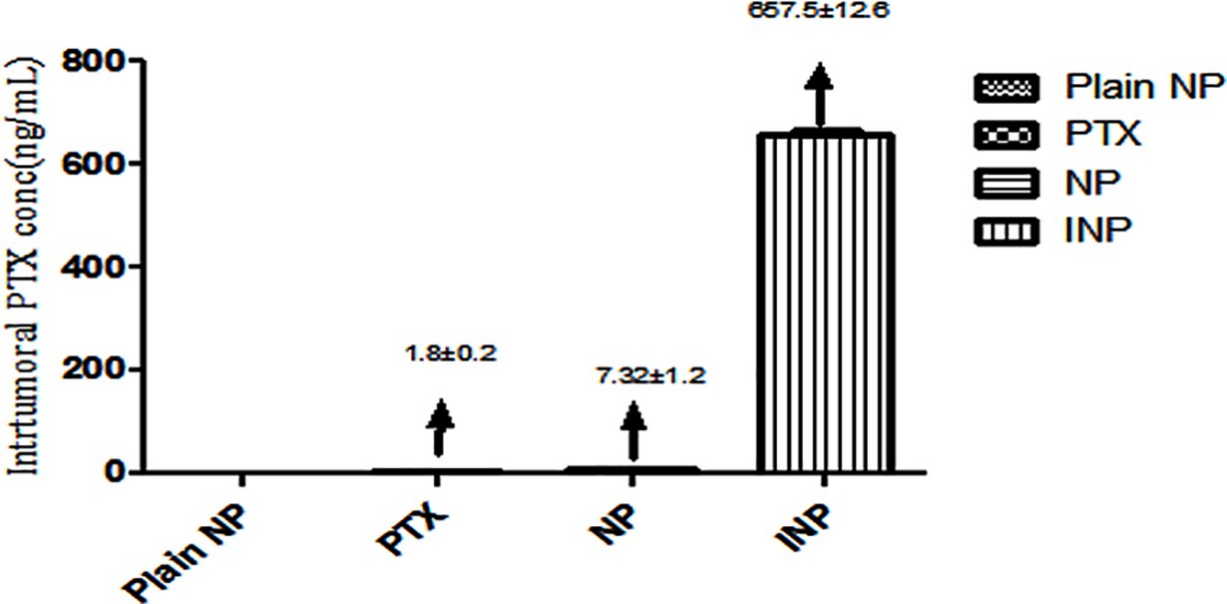
Plasma PTX concentration in the athymic female mice tumors treated with plain NP, PTX, NPs, INP and the PTX concentration was calculated by RP-HPLC method. The INP showed 93 fold higher tumors PTX concentration than NPs.

To analyse the effect PTX distribution on the animal plasma, the whole blood was collected from the mice. The plasma PTX concentration was calculated by RP-HPLC method at different time interval upto 24 h. As the (Fig 12) showed, plasma PTX concentrations were higher in mice treated with INP containing anti-EGFR targeting protein than NPs treated group. Statistical analysis was done in between INP and PTX solution as well as between NP and PTX at all the time points. These results finding showed that, INP have significant PTX concentration than PTX loaded nanoparticle and PTX treated groups. Since nanoparticle have a longer circulation than PTX formulations. INP have been shown comparatively more negative surface charge (zeta potential) than NP, with values of -3.48 mV, which increases the accumulation of PTX in the plasma and induces the longer circulation half-life. The plasma concentration of INP treated group showed 93 fold increased concentration than NP (Fig 13). Higher amount of PTX accumulation of INP in tumor was observed due to longer circulation of PEGylated nanoparticle without phagocytosis action and enhanced permeability as well as anti-EGFR protein specifically bounded with EGFR receptor expressed by TNBC.

### 3.5. Bio-distribution and targeting efficiency

**Fig 14:**
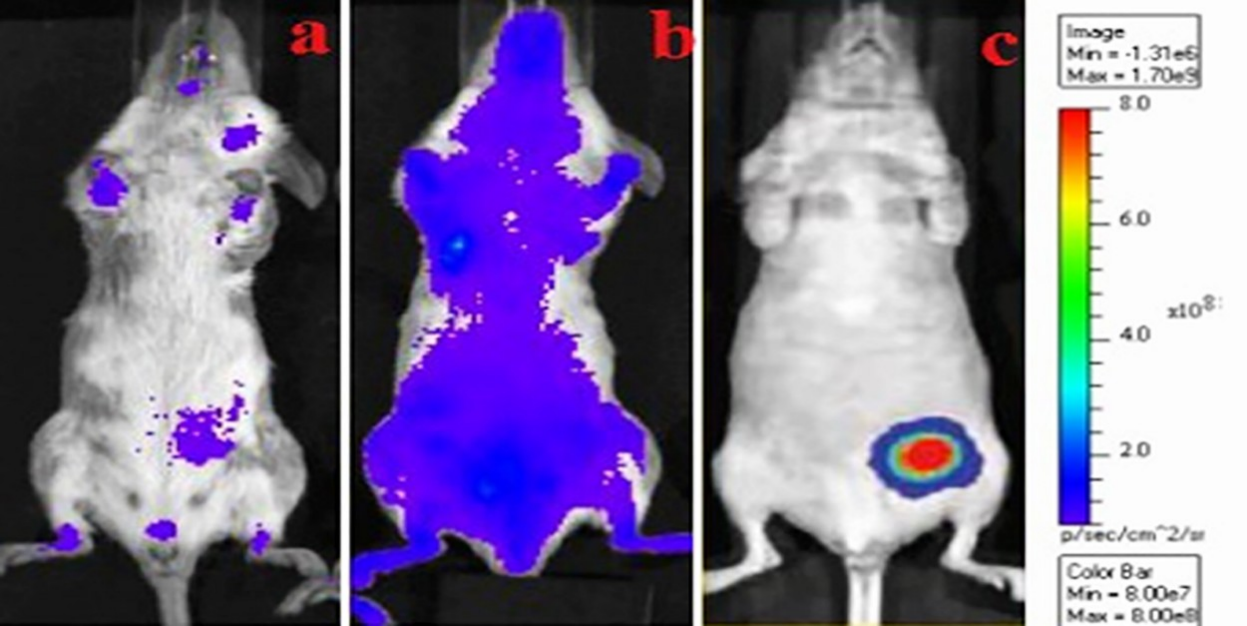
*In-vivo* fluorescence imaging studies for tumor cells target. a) PTX treated mice b) PTX-NP treated mice c) anti-EGFR targeted PTX loaded immunonanoparticle. The TNBC cells were specifically targeted by anti-EGFR anchored immunonanoparticle containing PTX than PTX nanoparticle and PTX treated animals. The fluorescence intensity and specific distribution were strong in in tumor vascular endothelial cells in case of anti-EGFR loaded immunonanoparticle than other treated animals.

The specific affinity of INP and PTX-NP were further analyzed by using an *in-vivo* fluorescence imaging system. After 24 hours of post-injection, the fluorescent emission intensities of the FITC-associated INP and PTX-NP were observed. The fluorescence imaging of tumors beared mice was shown in (Fig 14); the images were compared with control (pure PTX). After 24 hours of treatment, the INP injected mice was shown high fluorescence intensity than PTX-NP and PTX injected mice.

## 4. Discussion

The NP was prepared by the nanoprecipitation method, hydrophobic PLGA polymers and paclitaxel were dissolved in a water-miscible solvent (DCM). Since, DCM is a class 2 type of solvent as per ICH guidelines, which are not suitable for human usage, but the usage was permitted under lower risk [17]. During the formulation of NPs, they are subjected to overnight stirring at room temperature, which could be evaporated maximum volume of DCM. The negligible amount remaining is not considered to be hazardous. The polymeric mixture was dispersed in water at low-frequency sonication to get monosized NPs, which produced more stable forms and with less aggregation.

Patrick Couvreur *et*.*al* investigated that NPs would be formed by Ouzo effect [18], stated that instantaneous nanoprecipitation involves complex interfacial hydrodynamic phenomena. The changes in the physicochemical properties along with that interface condition as well as mechanical mixing between the two unequilibrated liquid phases, reimburse the discrepancies in free energy. Thus, the unforced mixing process results in polymer partition in the aqueous phase, and then it is aggregated like colloidal polymer particles upon solvent displacement [19].

The TEM images (Fig 1) shows NP shape and surface morphology, which are spherical in shape with smooth surfaces. When NPs are injected into the biological system or cellular fluid, the particles were quickly surrounded by biosystem macromolecules, which modify all the biological properties and gets eliminated from the human body by opsonin action. In order to avoid the elimination, NPs are coated with an anti-opsonin polymer like polyethylene glycol (PEG) and the NP had improved stability in the water by reducing the aggregation action [20].

As per the drug release studies, initially, the formulation showed burst drug released around 7%. From this, we observed that the low drug release is due to formation of the polymer layer around the drug and the drug molecules were crosslinking between the lacto-glycolic acid linkages which resulted in the sustained release of drugs for an extended period of time [21]. At the end of the 48 h, the narrow percentage of the drug was released from NP and INP 85.14 ± 1.2%, 80.24 ± 3.2% respectively. The coupling of anti-EGFR, does not affected the drug releases and the kinetics of drug release of NP and INP followed the zero order followed by Higuchi type of PTX release. The kinetic model expressed that both the formulations significantly followed the sustained action under diffusion controlled release from the matrix of the nanoparticle. From the above release studies, we could confirm that both the formulations shows effective sustained drug release and it may give optimal bioavailability.

NPs were able to target the TNBC cells by crafting with the anti-EGFR on the surface. The monoclonal antibody must possess the biological action of targeting ligand. Kim *et*.*al*, reported that anti-EGFR loaded nanoparticle was prepared with PAMAM-PLA-OH [22] and Nahta *et al* worked on anti-HER-2 mAb on PLGA NP by two different mechanistic of covalent and non-covalent bonding procedure [23]. The anti-EGFR was covalently attached to the surface of the NP by using EDC as a cross-linking agent. The direct coupling of anti-EGFR with the surface of NP by covalent bonding of carboxylic group and amino groups in EGFR protein and PLGA polymeric functional group. The integrity of monoclonal antibody may be diminished by covalent bonding action. SDS-PAGE analysis proved that anti- EGFR protein integrity was not destroyed by coupling action.

The cytotoxicity of pure paclitaxel and NP and INP were characterized by cancer cell viability by subject, to MTT assay method using MDA-MB-468 breast cancer cells. Initially, the IC_50_ value of PTX was determined by subjecting cancer cells to various concentrations of PTX and showed 2.27 μg/ml. The normal range of concentrations of PTX (0.025 to 20 μg/ml) corresponds to plasma levels of the drug reachable in humans [24]. The results were compatible with other published studies and the NPs showed increased cytotoxicity as compared to PTX, showing the smaller IC_50_ over PTX.

Based on the IC_50_ values of PTX, the NPs dose was fixed for further evaluations. The cell growth inhibition was higher when incubating for longer periods of time (48 h). Similar results were observed in the previous report in cell cytotoxicity of PTX in various human tumor cell lines like breast carcinoma (MCF-7), carcinoma cervical (HeLa) [25], laryngeal cancer Hep-2 cells [26] and lung cancer cell line (A549) [27] and its cytotoxicity was more significant than that of free paclitaxel. The above results suggest the IC_50_ concentration could be sufficient to maintain the safe therapeutic concentration of PTX over a prolonged period of time to provide effective therapeutic actions, without affecting normal cells.

The cytotoxic activity of NP and INP were compared with pure paclitaxel and plain NP at 24 and 48 h incubation. The sustained activities of NPs showed the biphasic release of incorporated PTX from the polymeric matrix of PLGA-PEG. The INP showed maximum sustained action by the mechanism of diffusion and erosion, which was reflected by the sustained activity of cytotoxic actions. The NPs and INP showed significant cellular uptake and confirmed by higher fluorescence emission from the cells. The images show FITC tacked NPs (Green) bound by the cellular constituents. The pictures clearly showed that NPs penetrated within the cell membrane boundary. The anticancer activity of NP depends on cytotoxicity and cellular uptake by cancer cells. Basically, NPs are non-specifically internalized into cells via endocytosis [28], broadly classified as phagocytosis and pinocytosis mechanisms. Pinocytosis is a favorable mechanism for small vesicle or particles uptake by cells, Pinocytosis can be happened by one of the following mechanism of uptake; clathrin‐mediated endocytosis, caveolin‐mediated endocytosis, macropinocytosis, and caveolin independent endocytosis action. These mechanisms are flexible based on specificity of cells and uptake particles [29].

The nuclear fragmentation which induced by the nanoparticles was observed by DAPI staining under a fluorescence microscope. The untreated MDA-MD-468 cells showed homogenous nuclei (smooth nuclear) with no evidence of segmentation and fragmentation. Whereas, cells treated with PTX-NP and INP were showed the apoptotic characteristic such as condensed chromatin, fragmented nucleus and formation of apoptotic bodies in MDA-MB-468 cells, which indicating that breakdown in the chromatin followed by DNA condensation. The nuclear condensation may occur due to the stress, it is the indication of cytotoxicity. The apoptotic and cytotoxicity induced by PTX-NP and INP were significantly different compared to control (PTX).

The EGFR targeted method is one of the optimistic strategies for TNBC treatment and successfully can improve the therapeutic efficacy in triple negative breast cancer patients. Yun Zhu *et al*., confirmed that EGFR gene expression in the TNBC patients (13–76%) [30]. Based on the evidence, the anti-EGFR could possibly target the TNBC cells without doubts. NP and INP treated TNBC cells expressed EGFR gene was identified and quantitatively measured by PCR analyzed by agarose gel electrophoresis. The PCR product was run on an agarose gel and a band showed an amplicon size of 270 bp. The INP treated cells shows, reduction in EGFR cDNA gene intensity than NP. The reduction of gene expression confirmed that anti-EGFR significantly targeted and bound with the specific EGFR gene.

The antitumor activity of EGFR protein target-specific antibody anchored nanoparticle (INP) showed significant anticancer activity by *in-vitro* model. The goal of the study was to examine the antitumor activity by Xenografts method using female nude mice, induced cancer by MDA-MB-468 cells to reach the tumor volume of 120 mm^3^. Animal treated with INP exhibit significant tumor volume reduction compared with NPs, PTX and plain nanoparticle. PTX solution drastically reduced the tumor volume initially. Later, the tumor volume was increased, due to the high elimination rate of PTX from the body. Furthermore, plasma isolated PTX was higher in INP treated group than NPs, due to anchoring of anti-EGFR on the surface of NPs and to target the EGFR protein expressed by TNBC cells. INP exhibit higher PTX accumulations (657.5±12.6 ng/ml) at 4 hours of post injection. However, satisfied therapeutic action was produced by the negatively charged and PEGylated nanoparticles showed less phagocytosis uptake, longer circulation time and sustained release [31]. Anti-EGFR anchored PTX loaded nanoparticle is very effective to reduce TNBC tumor volume in the Xenografts animal model. The targeting efficiency of INP compared with PTX-NP and PTX treated animals. All the three formulations were conjugated with FITC as the fluorescence probe. The animal images were observed under the *in-vivo* fluorescence imager system. The INP shown high tumor distribution, specific targeting efficiency and high fluorescence intensity than PTX and PTX-NP treated groups. Jing du *et al*., investigated that NP loaded anti-VEGFR2 and HER2 dual targeted showed significant targeting than single receptor. Since, the INP could specifically target strongly on TNBC vascular endothelial cell than PTX-NP and PTX. The PTX-NP has no target efficiency and distributed to all the parts of the body including tumor endothelial cells. The imaging studies expressed that, PTX-NPs and PTX distributed all over the body and it produces the toxicity. *In-vivo* imaging study recommended that anti-EGFR anchored PTX-NP showed significant specific targeting efficiency on the TNBC cells.

## 5. Conclusion

TNBC is unable to be treated by chemotherapy, due to lack of specific receptors on the cell surface. We tried to target the TNBC cells by focusing other protein expressions. The NP encapsulated PTX and anchored anti-EGFR, resulting in average NP range that internalized by MDA-MB-468 cells. Treatment with these INP showed significant cytotoxicity compared with NP and PTX. Moreover, the INP significantly reduces the expression of EGFR protein and target specifically TNBC cells compared with NP and PTX. Our results showed evidences that, anti-EGFR anchored PTX loaded nanoparticle may be able to deliver therapeutic drug to the TNBC cells.
